# Recuperación de espermatozoides de la orina en hombres con eyaculación retrógrada

**DOI:** 10.1515/almed-2023-0151

**Published:** 2024-07-12

**Authors:** Ernesto Veiga Álvarez, Nuria Zopeque García, Javier M. Gutiérrez Romero, Pilar Reimundo Díaz-Fierros, María D. Lozano Arana, Tamara Rodríguez Pérez, Javier Sánchez Álvarez, Guadalupe Bueno Rodríguez, Vanesa Castañón Bernardo, María J. Moyano Gallego

**Affiliations:** Comisión de Andrología y Técnicas de Reproducción Asistida, Sociedad Española de Medicina de Laboratorio (SEQC-ML), Barcelona, España; Unidad de Reproducción Humana Asistida, Laboratorio Central, Hospital Clínico Universitario de Santiago de Compostela, Santiago de Compostela, España; Laboratorio de Reproducción Humana Asistida, Servicio de Análisis Clínicos, Hospital Universitario Fundación Alcorcón, Alcorcón, Madrid, España; Laboratorio de Reproducción Humana Asistida, Servicio de Análisis Clínicos, Hospital Universitario Puerta del Mar, Cádiz, España; Laboratorio de Reproducción Asistida y Andrología, Servicio de Bioquímica Clínica, Laboratorios Clínicos Vall d’Hebron, Hospital Universitario Vall d’Hebron, Barcelona, España; Laboratorio de Reproducción Humana Asistida, UGC de Medicina Materno Fetal, Genética y Reproducción, Hospital de la Mujer, Hospital Universitario Virgen del Rocío, Sevilla, España; Laboratorio de Andrología y Técnicas de Reproducción Asistida, Servicio de Análisis Clínicos, edificio maternal, Hospital Universitario La Paz, Madrid, España; Laboratorio de Reproducción Humana Asistida y Andrología. Servicio de Análisis Clínicos, Hospital Universitario Virgen de Valme, Sevilla, España; Laboratorio de Reproducción Humana Asistida, Hospital Universitario Central de Asturias, Oviedo, España; Laboratorio de Reproducción Asistida; UGC Análisis Clínicos, Hospital Universitario Reina Sofía, Córdoba, España

**Keywords:** aspermia, azoospermia, eyaculación, infertilidad masculina, semen, disfunción sexual

## Abstract

**Introducción:**

La eyaculación retrógrada (ER) es la ausencia total o parcial de emisión del esperma, con la consecuente derivación del semen hacia la vejiga durante la fase de emisión de la eyaculación. Existe un grupo de pacientes en los que la evaluación del eyaculado no es suficiente para su detección. Y, en ocasiones, va a ser necesario recurrir a la utilización de métodos invasivos como la extracción de fluido epididimario, o la biopsia testicular para tratar su infertilidad.

**Contenido:**

El documento define la ER, y los métodos para su diagnóstico. También aborda la detección de espermatozoides en la orina poseyaculado (OPE), la preparación y recuperación de espermatozoides de la orina, y su uso posterior en técnicas de reproducción asistida.

**Resumen:**

El diagnóstico de ER se basa en la detección de espermatozoides en OPE en pacientes con aspermia o con oligozoospermia y volumen seminal bajo o normal. Aunque la presencia de espermatozoides en OPE podría considerarse por sí misma diagnóstica de ER, existe una falta de consenso en cuanto a definir exactamente los criterios diagnósticos y son muy pocos los estudios que los describen. Un correcto diagnóstico de la ER permite utilizar OPE para la obtención de espermatozoides y su posterior uso en técnicas de reproducción humana asistida, evitando técnicas invasivas.

**Perspectivas:**

Un importante número de pacientes con ER puede permanecer sin diagnosticar. Por ello, es fundamental realizar el estudio de ER en pacientes con sospecha, mediante el estudio de la OPE, siendo necesaria una adecuada interpretación de los resultados para su correcto diagnóstico.

## Introducción

Durante la eyaculación, el esfínter interno del cuello de la vejiga (EICV), debe permanecer cerrado para evitar una eyaculación retrógrada (ER), consistente en el retroceso del flujo, parcial o total de semen, hacia la vejiga [1].

Entre las causas más comunes de ER se incluyen las cirugías que producen incompetencia del cuello de la vejiga, el uso de medicamentos, la radioterapia para tratar el cáncer en el área pélvica, la esclerosis múltiple, la disección de los ganglios linfáticos retroperitoneales, la lesión de la médula espinal, la neuropatía secundaria en pacientes con diabetes mellitus (DM), o bien causas idiopáticas [2], [3], [4], [5], [6], [7], [8], [9], [10], [11], [12], [13], [14], [15], [16], [17].

La ER típica se caracteriza por aspermia seguida de micción con orina turbia, aunque también se presenta con hipospermia (volumen de eyaculado <1,4 mL) y oligozoospermia, y en ocasiones puede observarse incluso un volumen seminal normal [4, 18], [19], [20]. Por otra parte, la presencia de hipospermia y azoospermia con conductos deferentes palpables puede deberse a una obstrucción del conducto eyaculador o, en ocasiones, a una disfunción eyaculatoria [21]. Aunque la presencia de espermatozoides en la orina poseyaculado (OPE) podría considerarse por sí misma diagnóstica de ER, no existe consenso en cuanto a los criterios exactos para distinguir una ER verdadera, del eyaculado retenido en la uretra que se observa con frecuencia en hombres sanos, y son muy pocos los estudios publicados sobre este tema [22], [23], [24].

El objetivo principal del presente estudio es revisar la bibliografía disponible sobre los distintos métodos que permiten establecer el diagnóstico de una verdadera ER. También, en base a la evidencia científica, recomendar cómo introducir en la práctica clínica el estudio de la OPE, y cómo aislar los espermatozoides de la misma para su uso en técnicas de reproducción humana asistida (TRHA).

## Fisiología de la eyaculación

La eyaculación es un proceso reflejo que consiste en la emisión y expulsión del semen fuera del cuerpo a través de la uretra [25]. En la emisión, los espermatozoides se impulsan desde el epidídimo hasta la uretra prostática debido a la contracción de los conductos deferentes, las vesículas seminales (VS), y la próstata. Durante este recorrido, los espermatozoides se mezclan con las secreciones de las glándulas prostáticas, bulbouretrales y de las VS. La uretra prostática se convierte en un compartimento a presión donde se acumula el semen, gracias a que su músculo liso interno y estriado externo, permanecen cerrados. Primeramente, se contraen la parte posterior de la uretra prostática y el cuello vesical, provocando la dilatación de la porción distal de la uretra prostática. Ello conlleva la acumulación de las secreciones prostáticas y de las ampollas deferenciales junto con los espermatozoides. A continuación, se contraen las VS desplazando el contenido acumulado en la uretra prostática, que constituirá la primera fracción del eyaculado, y aportando la secreción más abundante del plasma seminal (aproximadamente el 70 %), que constituirá la fracción final [1].

En la expulsión, el incremento de presión en la uretra prostática junto con la contracción intermitente de los músculos bulbo e isquiocavernoso, que rodean la base del pene y sincronizados con los músculos colaterales del suelo pélvico, provocan la apertura del esfínter externo de la uretra prostática permitiendo la eyaculación de manera anterógrada (EA). Hasta que finaliza esta fase, el cuello vesical y el esfínter interno de la uretra prostática permanecen contraídos para evitar un retroceso del eyaculado.

La eyaculación, desencadenada por la estimulación mecánica del pene, requiere de la coordinación del SN simpático (controla el cierre del EICV), parasimpático y somático. Además, el cerebro ejerce una función moduladora que permite inhibir o estimular el reflejo eyaculatorio [1, 2, 26], [27], [28].

### Definición de eyaculación retrógrada

La ER es un tipo de disfunción eyaculatoria postesticular en la que tras el orgasmo parte o la totalidad del semen es propulsado hacia la vejiga urinaria debido a un mal funcionamiento del EICV. Puede ser completa, con ausencia de eyaculado (aspermia), o parcial en la que se observa un eyaculado de aspecto aparentemente normal pero con una disminución del volumen seminal (hipospermia) y de la concentración de espermatozoides, así como presencia de espermatozoides en la OPE [3, 18, 29].

### Prevalencia

Es una causa poco frecuente de infertilidad, siendo responsable del 0,3–2 % de los casos de infertilidad masculina [30], pero está implicada en el 14–18 % de los pacientes con aspermia [31, 32].

### Causas

Se incluyen causas anatómicas, farmacológicas, endocrinas y neurogénicas [2], [3], [4], [5], [6], [7], [8], [9], [10], [11], [12], [13]. Entre las más comunes están el uso de medicamentos (alfa bloqueantes, antidepresivos, antipsicóticos, bloqueantes ganglionares), las anomalías anatómicas congénitas o las debidas a cirugías que producen incompetencia del EICV (prostatectomía transuretral, cistectomía, lesión vesical traumática …), el desarrollo de tumores y los tratamientos de radioterapia en el área pélvica [14]. Dentro de las causas neurogénicas destacan los trastornos neurológicos con pérdida de la inervación simpática en el cuello de la vejiga como la esclerosis múltiple, la disección de los ganglios linfáticos retroperitoneales sin preservación de los nervios o la lesión de la médula espinal, que conducen a una incompetencia funcional del cierre del cuello de la vejiga [15]. Así mismo, otra causa neurogénica frecuente de ER es la neuropatía secundaria en pacientes con DM mal controlada, que provoca fallo del SN simpático y consecuentemente del cierre del EICV, con una prevalencia del 6–34 % en función de las diferentes poblaciones y estudios [16, 17]. Aun así, parece que la causa idiopática es la etiología más común en la ER parcial (82 %) [15].

### Diagnóstico

Ante la presencia de aspermia, o la detección de hipospermia asociada a oligozoospermia, se debe sospechar la posibilidad de ER [33, 34]. Vroege y col. [35]. sugirieron que el análisis y la confirmación de espermatozoides en OPE apoya el diagnóstico de ER aunque no de forma definitiva. Por otra parte, la detección espectrofotométrica de fructosa en OPE, mediante su reacción colorimétrica (amarillo-naranja) con el indol, ayuda a confirmar la presencia de ER [18, 36], [37], [38]. Hay que tener en cuenta que la fructosa está presente de forma natural en frutas y verduras y comúnmente se añade a alimentos y bebidas procesadas, miel y jarabes [39]. Los niveles normales de fructosa detectados en la orina de hombres adultos mediante técnicas muy sensibles como la cromatografía líquida de alta resolución acoplada a detector de espectrómetro de masas en tándem (UPLC-MS/MS, *ultra-performance liquid chromatography-mass spectrometry*), incluso tras altas ingestas de fructosa, se sitúan de no detectables a 0,5298 mmol/L [39, 40]. Teniendo en cuenta que la concentración de fructosa en el eyaculado es más de 25 veces la de la orina, siendo de media 14,2 mmol/L en hombres con paternidad probada (HPP) [41] y de 13,5 mmol/L en hombres a estudio de infertilidad (HEI) [42], la presencia de concentraciones de fructosa en OPE superiores a los niveles basales, por el paso de semen a la orina, servirán de apoyo diagnóstico a la ER.

Aunque el criterio diagnostico se establece por la presencia de espermatozoides en OPE [20], está demostrado que esto también ocurre en el 60–70 % de HPP [22], [23], [24], lo que cuestiona dicho criterio [43]. Mehta y col [44]. demostraron que la mayoría de estos espermatozoides se encuentran en la primera fracción de la orina. Así, muchos investigadores sugieren que una causa común de OPE positiva es el semen retenido en la uretra en lugar de una verdadera ER [29]. Para evitar falsos positivos en el diagnóstico de ER, se han desarrollado nuevos métodos diagnósticos como la aspiración suprapúbica vesical tras el orgasmo, donde la presencia de espermatozoides tanto en OPE como en la orina recogida de la vejiga preeyaculado (con mayor concentración observada en OPE) supondría una ER verdadera, mientras que su presencia únicamente en OPE supondría una retención de semen en la uretra [34]. Otra alternativa es monitorizar la eyaculación en tiempo real mediante ecografía transrectal, siendo posible visualizar si el cuello de la vejiga se mantiene abierto durante la emisión y la expulsión [45, 46].

### Tratamiento

Antes de iniciar cualquier terapia, deben descartarse posibles etiologías reversibles como el consumo de fármacos directamente relacionados con la aparición de ER.

El tratamiento es especialmente importante para aquellos pacientes con ER y deseo genésico.

La farmacoterapia y las intervenciones quirúrgicas dirigidas al tratamiento de la ER son limitadas [43]. La bibliografía se limita a series pequeñas y ensayos aleatorizados por lo que se necesitan más estudios y ensayos controlados aleatorizados [33].

No obstante, en ausencia de etiologías corregibles (anomalías anatómicas, tumores, diabetes), el tratamiento de primera línea es el farmacológico. Su objetivo es aumentar el tono simpático tanto del EICV como de los conductos deferentes, evitando el flujo retrógrado de semen hacia la vejiga mediante la estimulación del SN simpático o la inhibición del parasimpático:

– Los fármacos simpaticomiméticos son especialmente útiles en pacientes con enfermedad progresiva lenta como la neuropatía diabética o en aquellos con falta de emisión debido a la interrupción de la inervación simpática retroperitoneal tras cirugía previa. Sin embargo, muchos de los estudios sobre su eficacia tienen un tamaño muestral pequeño y resultados no concluyentes [33]. Los más comúnmente utilizados son sinefrina, clorhidrato de pseudoefedrina, efedrina, fenilpropanolamina y midodrina. Estos pueden conseguir aumento del volumen eyaculatorio y resolución de la ER en pacientes aspérmicos permitiendo que las parejas conciban de forma natural [47, 48]. Desafortunadamente, presentan efectos secundarios como diversos grados de mareos, alteraciones del sueño, debilidad, inquietud, sequedad de boca, náuseas o sudoración, que ocurren frecuentemente en los pacientes respondedores; y, además, taquicardia e hipertensión, por lo que deben utilizarse con precaución en pacientes diabéticos con riesgo de enfermedad cardiovascular [33, 48], [49], [50], [51].
– El uso aislado de parasimpaticolíticos, como el maleato de bromfeniramina e imipramina, presenta una eficacia del 22 % frente al 39 % cuando se usan en combinación con simpaticomiméticos [52]. Así, la terapia combinada parece más efectiva, aunque el análisis estadístico no es robusto debido a los pequeños tamaños muestrales [33].
– Como opciones alternativas se encuentran el uso de buspirona [53] o la inyección transuretral de colágeno o Deflux^®^ (gel viscoso, biodegradable, biocompatible y no migratorio, formado por microesferas de dextranómero y ácido hialurónico estabilizado de origen no animal), que puede restablecer el sentido anterógrado de la eyaculación [43].

Finalmente, pese a que las intervenciones quirúrgicas resultan exitosas, solo se han descrito dos pequeñas series de casos hace ya cuatro décadas [52]. Por ello, antes de su aplicación, se deben sopesar otras opciones como el uso de espermatozoides obtenidos de OPE o del testículo para uso posterior en TRHA [14].

### Manejo clínico

Una vez confirmada la ER en pacientes con deseo genésico, y en caso de ineficacia de los diferentes tratamientos propuestos, la alternativa no quirúrgica más frecuente, efectiva y económica en TRHA es la recuperación de espermatozoides viables de OPE [9, 13]. La adecuada obtención de la OPE y su rápido procesamiento, permitirán preservar la viabilidad de los espermatozoides.

Se han descrito tres métodos de recuperación de espermatozoides de OPE [2, 3, 9, 13, 15, 33, 52]:

1. Eyaculación con vejiga llena: Su objetivo es conseguir un EA ya que la vejiga llena puede evitar el paso del eyaculado coagulado a la misma. Asimismo, esto puede favorecerse si la masturbación se realiza en bipedestación [54]. Aun consiguiendo un EA, se ha demostrado que parte del eyaculado todavía sigue entrando en la vejiga, y que cuando existe una disfunción severa del EICV no se consigue un EA [54]. En caso de no EA, se analizaría la OPE para confirmar si hay espermatozoides o no en la orina [54, 55].
2. Procesamiento de OPE alcalinizada: El objetivo es neutralizar la acidez de la orina para mantener la viabilidad de los espermatozoides presentes en OPE. Primeramente, la orina se alcaliniza (mediante la ingestión de bicarbonato sódico, acetazolamida, o citrato de potasio) o bien se diluye aumentado la ingesta de fluidos. Seguidamente, se obtiene la OPE por micción o por cateterismo y se centrifuga [14, 52, 56, 57]. El sedimento obtenido se resuspende en medio de cultivo, suplementado habitualmente con albúmina [5, 57], y los espermatozoides así aislados se emplean para auto inseminación vaginal, inseminación intrauterina (IIU), fecundación *in vitro* convencional (FIV) o inyección intracitoplasmática de espermatozoides (ICSI).
3. Método Hotchkiss: Consiste en vaciar la vejiga antes de la eyaculación mediante un catéter uretral para posteriormente realizar un lavado de la misma con solución lactato de Ringer reduciendo así el ambiente ácido de la vejiga. Posteriormente, se retira el catéter, el paciente eyacula y el contenido de la vejiga se vacía mediante micción o nuevo cateterismo [43, 58]. La técnica Hotchkiss modificada consiste en instilar en la vejiga medio de cultivo estéril para espermatozoides conteniendo albúmina [59, 60]. Los espermatozoides así obtenidos se utilizan para TRHA.

Finalmente, si la recuperación de espermatozoides de OPE falla, es posible recurrir a alternativas quirúrgicas como la obtención de espermatozoides del epidídimo (PESA: *Percutaneous epididymal sperm aspiration*) o del testículo (TESE: *Testicular sperm extraction*) [2]. Es recomendable empezar por el más sencillo y menos invasivo de los métodos posibles y, en caso de no tener éxito, pasar progresivamente al siguiente método en complejidad e invasividad.

### Procesamiento de la OPE

#### Fase preanalítica

##### Preparación del paciente

Los rangos fisiológicos de pH y osmolalidad del semen eyaculado son 7,2–8,2 y 300–380 mOsm/kg, respectivamente [18]. Debido a su diferente pH y osmolalidad, la orina puede lesionar la membrana plasmática, el acrosoma y la pieza intermedia de los espermatozoides y alterar su motilidad y parámetros cinéticos [15, 61, 62]. Las alteraciones de la osmolalidad (hipoosmolalidad especialmente ya que las soluciones hiperosmolares mantienen perfectamente la motilidad) y la presencia de compuestos nitrogenados (NH4^+^) producen efectos más acusados que alteraciones del pH [5, 31, 33, 59, 63], [64], [65], [66], [67]. Así pues, se debe prestar especial atención a la preparación del paciente con ER antes de la recogida de las muestras de semen y OPE (Tabla 1).

**Tabla 1:** Instrucciones de recogida de muestra de semen y orina poseyaculado para uso posterior de los espermatozoides en TRHA.

De 2 a 7 días previo a la recogida	– Abstinencia sexual, siendo la recomendación 3 días
Día previo a la recogida	– Abstinencia sexual
	– Beber como mínimo 1,5 litros de agua repartidos a lo largo del día
	– Evite los alimentos ácidos la noche anterior: Alcohol, refrescos, carnes grasas, alimentos fritos, vinagre, tomates, frutas cítricas (limón, naranja, pomelo, kiwi), pan de harinas refinadas, azúcar, yogures, cacao
	– Tome alimentos antiácidos la noche anterior: Patatas al vapor, aguacate, rabanitos, calabaza, brócoli, guisantes, pepino, pescado y carnes blancas, plátano, papaya, leche, avena, manzanilla
	– Tomar una cucharada sopera de bicarbonato sódico (2,5 g) disuelta en un vaso de agua (250 mL) 15 minutos después de la cena
Día de la recogida	– Al levantarse, orinar con el fin de reducir lo máximo posible el volumen de orina de la vejiga
	– Desayunar como lo hace habitualmente unas 2 horas antes de acudir al laboratorio, pero evitar el café, té, los alimentos ácidos y el tabaco
	– A los 15 minutos de acabar el desayuno tomar dos cucharadas soperas de bicarbonato (aproximadamente 4–5 g) disueltas en dos vasos de agua (500 mL) una hora antes de iniciar la recogida. En diabéticos disolver en 3 vasos de agua, 750 mL
	– Acudir al laboratorio a la hora programada sin haber vuelto a orinar, donde se le entregarán dos recipientes estériles de recogida de muestra de boca ancha:
	* Uno para intentar recoger muestra de semen mediante masturbación: Rotulado como “Semen”
	* Otro con 10 mL de medio de lavado de semen para recoger la orina poseyaculado: Rotulado como “Orina”
	– Antes de la masturbación, volver a orinar con el fin de reducir lo máximo posible el volumen de orina de la vejiga^a^
	– Posteriormente, masturbarse de pie y recoger el semen, si hubiera eyaculación, en el recipiente estéril e identificado como “Semen”.
	– Después de la eyaculación, y tan pronto como sea posible, recoger una muestra de orina en el frasco rotulado como “Orina”, distinto al de semen y que contiene 10 mL de medio de lavado, llenando sólo la cuarta parte del mismo (marca de 25 mL).
	– Entregar en el laboratorio lo más rápidamente posible ambas muestras para su procesamiento inmediato.

^a^En caso de que se recogiera esta orina para medir pH y osmolalidad, el pH debería oscilar entre 7,6 a 8,1 y la osmolalidad entre 300 y 500 mOsm/kg de solución.

##### Recolección de la muestra de semen/OPE (propuesta de la comisión de andrología y técnicas de reproducción asistida de la SEQC^ML^)

Las recomendaciones a seguir para la obtención de muestras de semen y para su análisis en un seminograma son las del manual de la OMS 2021 [18]. En caso de sospecha de ER tras el análisis de la muestra o de no obtención de la misma (orgasmo seco), sugerimos programar una nueva recogida de semen con la vejiga llena y en bipedestación y, en caso de obtener EA, valorar la muestra de semen. El hecho de obtener así EA, u observar una mejoría en los resultados obtenidos, permite plantear a los pacientes la opción de mantener relaciones teniendo el varón la vejiga llena, o bien de recoger EA con la vejiga llena y en bipedestación para su posterior uso en TRHA [54]. Si aun así no se obtiene un EA, recomendamos recoger la OPE y analizarla para determinar en ella la presencia de espermatozoides. En caso afirmativo, se programará una tercera recogida de muestra y se proporcionarán instrucciones para alcalinizar previamente la orina con bicarbonato de sodio vía oral y para diluirla aumentando la ingesta de fluidos (Tabla 1) [68].

Esta tercera recogida se realizará siempre en el laboratorio. Primeramente, el paciente debe orinar y vaciar completamente su vejiga. En esta orina previa a la masturbación, será necesario determinar si el pH y osmolalidad están en rango óptimo, en caso contrario será necesario continuar tomando bicarbonato. Tras confirmar datos óptimos de pH y osmolalidad en la orina, intentar obtener un EA en un recipiente de recolección estéril, o al menos llegar al orgasmo en casos de aspermia. Rápidamente después, el paciente debe orinar entre 10–50 mL de orina (como máximo hasta la mitad del frasco) en otro recipiente estéril que contenga en torno a 10 mL de medio de cultivo para lavado de espermatozoides (ayuda a mantener el pH y la osmolalidad del semen presente en la orina), precalentado a 37 °C y suplementado con albúmina sérica humana, y entregar ambas muestras al personal de laboratorio en un plazo máximo de cinco minutos tras su recogida [15, 69].

#### Fase analítica

Para una correcta evaluación de la ER, es imprescindible que el paciente entregue una OPE y, en caso de haber conseguido EA, una muestra de semen para su análisis [15, 70].

##### Consideraciones técnicas en relación al análisis microscópico de las muestras de OPE

Para la valoración de las muestras de orina, los objetivos de aumento 10× se consideran de baja magnificación (LPF, *low power microscopy field*) mientras que los de 20× y 40× se consideran de alta magnificación (HPF, *high power microscopy field*). La reciente norma UNE-EN ISO 23162:2022 aunque aconseja el uso tanto de 20× como 40× para la evaluación de la ausencia de espermatozoides (apartado 6.3.6) sólo define como HPF a los de 40× [71]. Por otro lado, el volumen de muestra observado en cada campo microscópico depende del área del campo (círculo) y de la profundidad de la preparación/cámara de contaje (ej. 20,7 µm para una preparación en fresco de 10 µL en portaobjetos y recubierta con un cubreobjetos de 22×22 mm, espesor #1,5 o #2). El radio del campo microscópico puede medirse con un micrómetro ocular o puede estimarse dividiendo el diámetro de la apertura de la lente ocular por la magnificación de la lente objetivo. Así por ejemplo, con un objetivo HPF de 40× y un ocular 10× de apertura 20 mm: cuatro espermatozoides por campo de visión corresponden a 1×10^6^ espermatozoides/mL, y con un objetivo HPF 20× y un ocular 10× de apertura 20 mm: 16 espermatozoides por campo de visión corresponden a 1×10^6^ espermatozoides/mL [18]. Con un ocular 10× de apertura 22 mm, la equivalencia sería de 5 y 20 espermatozoides por campo de visión respectivamente (Tabla 2).

**Tabla 2:** Equivalencia en millones/mL de los espermatozoides observados/campo de visión microscópica en función de la lente ocular y lente objetivo, observados con una profundidad de campo de la preparación de 20,7 µm (10 µL de muestra depositada en portaobjetos y cubreobjetos de 22×22 mm^a^).

Lente ocular 10× +lente objetivo 20×						
	Diámetro del campo^b^	Radio del campo (r)	r^2^	Área del campo (A)^c^	Volumen del campo^d^	Número de espermatozoides/campo^e^
Apertura ocular 20 mm	1 mm	500 µm	250.000 µm^2^	785.500 µm^2^	16.259.850 µm^3^≈16×10^−6^/mL	≈16
Apertura ocular 22 mm	1,1 mm	550 µm	302.500 µm^2^	950.332 µm^2^	19.671.872 µm^3^≈20×10^−6^/mL	≈20

**Lente ocular 10× +lente objetivo 40×**						

	**Diámetro del campo^b^**	**Radio del campo (r)**	**r^2^**	**Área del campo (A)^c^**	**Volumen del campo^d^**	**Número de espermatozoides/campo^e^**

Apertura ocular 20 mm	0,5 mm	250 µm	62.500 µm^2^	196.375 µm^2^	4.064.962 µm^3^≈4×10^−6^/mL	≈4
Apertura ocular 22 mm	0,55 mm	275 µm	75.625 µm^2^	237.583 µm^2^	4.917.968 µm^3^≈5×10^−6^/mL	≈5

^a^Cubreobjetos de espesor #1,5 – 0,16 a 0,19 mm – o #2 – 0,19 a 0,23 mm –. ^b^Diámetro del campo=apertura ocular en mm/magnificación del objetivo. ^c^Área del campo (A)=πr^2^. ^d^Volumen del campo=A×profundidad campo. ^e^Número de espermatozoides/campo equivalentes a 1×10^6^/mL.

Para la evaluar la presencia de espermatozoides en OPE recomendamos:

– Usar una lente ocular de 10× (apertura 22 mm).
– Dispensar una alícuota de 10 µL entre un portaobjetos y un cubreobjetos de 22×22 mm (espesor #1,5 – 0,16 a 0,19 mm – o #2 – 0,19 a 0,23 mm –) que permitirá la dispersión completa de la muestra.

##### Procesamiento y análisis de las muestras de eyaculado y OPE para TRHA (propuesta de la Comisión de Andrología y Técnicas de Reproducción Asistida de la SEQC^ML^)

Si se obtiene EA se procede a su análisis habitual según los últimos criterios del manual de la OMS 2021 [18].

**Figura 1:**
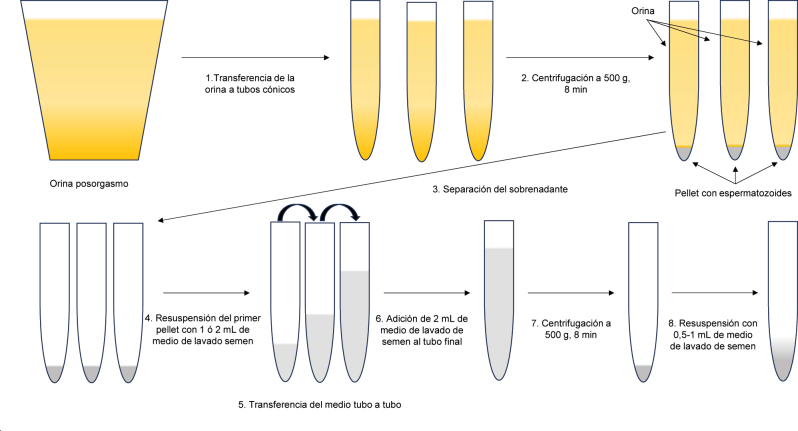
Aislamiento de los espermatozoides de la OPE.

Por otro lado, se procede a recoger y evaluar la OPE determinando pH y osmolalidad en una alícuota de la misma. Seguidamente, se analiza en busca de espermatozoides [15, 70, 72]. Es importante destacar que los espermatozoides de OPE son extremadamente frágiles debido a que la orina puede lesionar la pieza intermedia [61] y deben manejarse con mucho cuidado [57]. Para el análisis primeramente, y nada más recibir la muestra en el laboratorio, se carga una cámara de contaje y se dispensa una alícuota de 10 µL entre un porta y cubreobjetos [71]. Seguidamente, se divide la OPE en alícuotas de 10 mL en tubos cónicos estériles, se anota el volumen total de orina y se centrifugan los tubos durante 8 min a 500 *g* [18] a temperatura ambiente, para separar cuanto antes los espermatozoides de la orina. Mientras se centrifuga, se evalúa de forma manual o automatizada la cámara de contaje y, en caso de observarse espermatozoides, se calcula su concentración y motilidad. Si no se detectaron espermatozoides en la cámara de contaje, se evalúan los 484 campos del portaobjetos. En caso de detectarse espermatozoides, se anota el número observado en cada campo y su motilidad. El resultado se expresará como una media, obtenida dividiendo el total de espermatozoides contados entre el total de campos observados [15, 70, 72]. Tras la centrifugación de los tubos de orina, se desecha el sobrenadante de cada uno sin perturbar el sedimento. Seguidamente, se reconstituye el sedimento del primer tubo con 2 mL de medio de lavado de espermatozoides, precalentado a 37 °C, y la mezcla se transfiere al segundo tubo para reconstituir su sedimento. Se repite el procedimiento sucesivamente hasta reconstituir todos los sedimentos. A la mezcla obtenida se le añaden otros 2 mL de medio y se centrifuga nuevamente durante 8 min a 500 *g* a temperatura ambiente. Tras la centrifugación, se desecha el sobrenadante sin perturbar el sedimento, y este se reconstituye con 0,5–1 mL de medio (en función de la concentración inicial y del pellet observado) y se anota el volumen final obtenido (Figura 1). Finalmente, se realiza un análisis manual o automatizado de la concentración y la motilidad espermáticas, al igual que se hizo con la muestra de OPE precentrifugación. Los espermatozoides así recuperados pueden ser utilizados directamente para su aplicación en TRHA, o bien ser criopreservados para su uso posterior [52, 72], [73], [74].

#### Fase posanalítica

##### Criterios para considerar un contaje de espermatozoides en OPE como positivo

Aunque la presencia de espermatozoides en OPE podría considerarse por sí misma diagnóstica de ER, existe una falta de consenso en cuanto a los criterios exactos para considerar un contaje de espermatozoides en OPE como positivo y son muy pocos los estudios publicados sobre este tema [4]. Los estudios hasta la fecha proponen tres criterios:

1. Seleccionar arbitrariamente un valor absoluto para el número de espermatozoides presentes en OPE, por encima del cual se clasificaría como positivo: Aunque no se han establecido criterios exactos, el hallazgo de más de 10–15 espermatozoides/HPF tras el centrifugado a 300 *g* durante 10 minutos de la muestra [2, 15, 21, 22, 44, 75], o bien, la presencia de más de un millón de espermatozoides totales en la muestra [76], son habitualmente considerados como criterio diagnóstico de ER. Hay que tener presente que los primeros trabajos donde se acepta este criterio se desarrollaron cuando los microscopios utilizaban lentes oculares de 20 mm pero que hoy en día se suelen montar de 22 mm.
  Por ello, la recomendación de la comisión de andrología y técnicas de reproducción asistida de la SEQC^ML^ como criterio diagnóstico, tras centrifugación de la OPE durante 8 min a 500 *g*, que es la recomendación dada en el actual manual de la OMS [18], es la presencia de 12–20 espermatozoides/HPF de 20× (0,625–0,94 millones/mL) que se corresponde a la equivalencia utilizando oculares de 22 mm.
2. Seleccionar arbitrariamente un valor para el porcentaje de espermatozoides presentes en OPE en relación al índice de retroeyaculación (IR). El IR se calcula dividiendo el número total de espermatozoides en OPE por el número total de espermatozoides eyaculados (que corresponde a su vez a la suma del número total en OPE más el número total en EA), y multiplicando el resultado obtenido por 100. Esta opción es solamente aplicable en caso de disponer de EA (la forma menos común de ER) y su planteamiento responde al rechazo de algunos autores a considerar la simple presencia de espermatozoides en OPE como condición suficiente para el diagnóstico de ER [77].
  La comisión de andrología y técnicas de reproducción asistida de la SEQCML acepta como criterio diagnóstico el percentil 97,5 propuesto por Ariagno y col [77] por el cual los HEI considerados retroeyaculadores serían aquellos cuyo número total de espermatozoides en OPE sea superior a 3,8×10^6^ y con un IR superior al 2,16 % [77].
3. Cuando el paciente es azoospérmico y presenta ER concomitante, no será posible detectar la presencia de espermatozoides en OPE para aplicar cualquiera de los dos criterios anteriores. En tal circunstancia, la detección de fructosa en una muestra de orina premasturbación (control negativo) y en OPE (test), permitirá diagnosticar la ER si hay presencia de fructosa en la OPE por encima de los niveles basales [5, 18, 33, 37, 38, 78]. Hay que advertir que en aquellos casos de azoospermia y agenesia de vesículas seminales no habrá fructosa en OPE no pudiéndose utilizar este criterio.
  Este mismo criterio puede aplicarse como tercer criterio diagnóstico de ER cuando existe EA. Así, se realizará la detección de fructosa en una muestra de orina premasturbación (control negativo), en EA (control positivo) y en OPE (test), La comisión de andrología y técnicas de reproducción asistida de la SEQC^ML^ propone como criterio diagnóstico valores de fructosa en OPE superiores a 0,53 mmol/L, ya sea determinada por el método del indol, enzimáticos o cromatográficos.

##### Dificultades prácticas en la aplicación de criterios para considerar un resultado en OPE positivo

La aplicación en la práctica clínica de cualquiera de los tres criterios propuestos resulta compleja, ya que la presencia de espermatozoides en OPE puede deberse a una verdadera ER en la vejiga o al eyaculado residual que permanece en la uretra y que posteriormente se lava con la micción; pudiendo este suceso variar intra e interindividualmente.

En hombres con EA, la mayoría de los espermatozoides presentes en la uretra tras la eyaculación se observan en la primera fracción de orina evacuada (generalmente entre 10–20 mL) y se eliminan con la primera micción [23]. Además, su probabilidad de detección es mayor cuanto mayor sea el número de espermatozoides presentes en el eyaculado y cuanto menor sea el tiempo transcurrido desde la eyaculación [23, 44, 79]. Así, se ha descrito que es posible observar espermatozoides en la mayoría de OPE obtenidas entre 0,5 y 4 h tras la eyaculación (en un 59,5 % de las OPE a los 30 minutos y en un 70 % a las dos horas, dejando de ser detectables tras cinco horas), y los últimos espermatozoides móviles se pueden encontrar en la OPE tras 4,5 horas de haber eyaculado [23]. Esto implica que, además de en los casos de aspermia, se debería estudiar la presencia de espermatozoides en OPE cuando el recuento de espermatozoides en el EA sea inferior a cinco millones/mL junto con un volumen inferior a 0,5 mL de semen en lugar de indicar directamente un tratamiento de ICSI, ya que podría tratarse de una ER susceptible de tratamiento o bien se podrían recuperar espermatozoides de OPE para realizar una IIU [44]. En cualquier caso, estos datos indican que la causa más común de OPE positiva se debe al lavado del semen retenido en la uretra en lugar de a una verdadera ER.

##### TRHA a realizar con los espermatozoides recuperados de OPE

La recuperación de espermatozoides en HEI con ER completa es impredecible y muy variable, por ello se aconseja criopreservar los espermatozoides recuperados en OPE de forma previa a la TRHA indicada [60].

En cuanto al procesamiento de los espermatozoides recuperados en OPE, ha sido descrito desde un simple lavado y centrifugación [54, 57, 80] hasta la aplicación de técnicas adicionales de selección como la centrifugación por gradientes de densidad [74, 80, 81] o el *swim up* [82], [83], [84]. Incluso podría lograrse el embarazo de forma natural tras haber realizado previamente una ICSI fallida gracias a la inyección transuretral de Deflux^®^ en el cuello de la vejiga, lo que restablece el EA [43].

## Conclusiones

Es necesario realizar el cribado de OPE en HEI con: Aspermia, azoospermia asociada a hipospermia, oligozoospermia asociada a hipospermia y en aquellos con historia de cirugía urológica previa o estenosis de la uretra distal.

Este es el primer trabajo que describe el criterio arbitrario de ER adaptado a lentes oculares de 22 mm. Así, la presencia de más de 12–20 espermatozoides/HPF con objetivo 20× y lente ocular de 22 mm, tras el centrifugado de la muestra de OPE, se podría considerar sugerente de ER. Igualmente, la presencia de fructosa en OPE es un parámetro adicional de apoyo al diagnóstico.

Por su sencillez, efectividad y naturaleza no invasiva, se recomienda la ingesta oral de un agente alcalinizante urinario tipo bicarbonato sódico para regular el pH urinario y su osmolalidad, optimizando así la muestra de OPE y el aislamiento de espermatozoides para uso posterior en TRHA. Además, hay varios productos de desecho excretados en la orina que pueden afectar a los espermatozoides, pero sus niveles pueden ser minimizados por la diuresis inducida antes de intentar la eyaculación y la recogida de OPE.

Se deben respetar las condiciones especiales descritas para la recolección de OPE y realizar una pronta micción poseyaculación, ya que esto aumenta la probabilidad de encontrar la mayor cantidad de espermatozoides vivos y móviles en OPE.

En aquellas parejas en las que el tratamiento farmacológico para la ER fracasa, la aplicación de TRHA mediante la recuperación de espermatozoides de OPE, o incluso directamente del epidídimo o del testículo, se presenta como la única alternativa para conseguir una gestación con gametos propios.
